# Optimal pain management: epidural or locoregional nerve blocks for canine stifle surgery?

**DOI:** 10.18849/ve.v11i2.736

**Published:** 2026-06-09

**Authors:** Tiffany Tang, Eduardo Uquillas

**Keywords:** ANALGESIA, CANINE, EPIDURAL INJECTION, LOCAL ANAESTHESIA, PAIN, STIFLE SURGERY

## Abstract

**PICO question:**

In dogs undergoing stifle surgery, do locoregional nerve blocks compared with epidural anaesthesia improve perioperative antinociception and reduce residual block?

**Clinical bottom line:**

**Category of research:**

Treatment.

**Number and type of study designs reviewed:**

Six studies were appraised; all were prospective, blinded, randomised, controlled clinical trials.

**Strength of evidence:**

Moderate.

**Outcomes reported:**

Overall, the studies suggest that locoregional nerve blocks are a viable alternative to epidural anaesthesia (EPID) for dogs undergoing stifle surgeries. All studies demonstrated consistent success with locoregional nerve blocks, while two studies reported failures with EPID. Moreover, all studies found that locoregional nerve blocks provided similar analgesic effects to EPID, with one study suggesting that EPID bupivacaine might offer better intraoperative analgesia but require more rescue analgesia compared to combined femoral and sciatic nerve block (F+S), while another study suggested that the saphenous and sciatic nerve block (SSNB) group had lower pain scores than the EPID group at 4 and 8 hours postoperatively. Additionally, one study suggested that F+S bupivacaine might lead to a lower incidence of urinary retention compared to EPID, while another study indicated that lumbar plexus and sciatic nerve block (LPS) with bupivacaine could facilitate an earlier return of motor function.

**Conclusion:**

Despite no conclusive evidence suggesting the superiority of either locoregional nerve blocks or EPID in dogs undergoing stifle surgery, there is evidence that locoregional nerve blocks may serve as an alternative to EPID, with a similar success rate, comparable perioperative analgesia, and fewer incidences of residual block.

How to apply this evidence in practice

The application of evidence into practice should take into account multiple factors, not limited to: individual clinical expertise, patient’s circumstances and owners’ values, country, location or clinic where you work, the individual case in front of you, the availability of therapies and resources.

Knowledge Summaries are a resource to help reinforce or inform decision making. They do not override the responsibility or judgement of the practitioner to do what is best for the animal in their care.

## Clinical scenario

You are a veterinary anaesthetist intern at a specialist referral centre preparing for the anaesthesia of a 30 kg male Labrador scheduled for a tibial-plateau levelling osteotomy (TPLO) next week. Upon reviewing recent research, you discover that epidural anaesthesia (EPID) has significant potential for side effects, such as urinary retention and motor impairment. Consequently, you decide to investigate alternative approaches, such as locoregional nerve blocks, which may offer similar analgesic efficacy with fewer complications.

## The evidence

Six prospective, blinded, randomised, controlled, clinical trials addressing the PICO question were reviewed (Bartel et al., 2016; Campoy et al., 2012; Caniglia et al., 2012; Graff et al., 2024; Kalamaras et al., 2021; McCally et al., 2015). The findings from these studies were consistent, providing weak-to-moderate evidence that locoregional nerve blocks provide similar analgesic efficacy to EPID, and weak-to-moderate evidence in reducing residual block instances compared with EPID. However, all included studies had methodological limitations. Overall, there is moderate evidence to suggest that locoregional nerve blocks can serve as a viable alternative to EPID in dogs undergoing stifle surgeries.

## Summary of the evidence

**Bartel et al. (2016):** Comparison of bupivacaine and dexmedetomidine femoral and sciatic nerve blocks with bupivacaine and buprenorphine epidural injection for stifle arthroplasty in dogs **Aim: **To compare intra- and postoperative analgesic efficacy and the incidence of residual block between femoral and sciatic nerve blocks (F+S) using bupivacaine and dexmedetomidine, and epidural anaesthesia (EPID) using bupivacaine and buprenorphine in dogs undergoing unilateral tibial plateau levelling osteotomy (TPLO).

Population:	Healthy client-owned dogs in the United States, ages ranging from 1 to 11 years (mean 5 years), weighing 36 ± 10 kg, undergoing elective unilateral TPLO.
Sample size:	26 dogs.
Intervention details:	Random allocation equally to one of two intervention groups: EPID group (n = 13) F+S group (n = 13) A sample size calculation determined that only 6 animals per group were needed to achieve 95% statistical power at a significance level of 0.05. Anaesthesia and medication administration: Dogs were premedicated with sodium citrate (20 mg/kg orally (PO)), acepromazine (0.02 mg/kg intramuscularly (IM) and hydromorphone (0.1 mg/kg IM) before induction with intravenous (IV) propofol, followed by maintenance with isoflurane in oxygen. At extubation, all dogs received carprofen (4 mg/kg subcutaneously (SC), followed by tramadol (3 mg/kg PO) 6 hours later, with additional doses every 8 hours for 24 hours. Administration of treatments 20 minutes after induction and prior to the final skin preparation for surgery: EPID: bupivacaine (1 mg/kg; bupivacaine HCl 0.5%) and buprenorphine (4 μg/kg) administered in a volume of 0.2 mL/kg. F+S guided by ultrasound: bupivacaine (0.5 mg/kg; bupivacaine HCl 0.5%) and dexmedetomidine (0.1 μg/kg) at each nerve. Intraoperative cardiovascular management: Phenylephrine infusion (0.5–1.0 μg/kg/min IV) was used for hypotension with normal HR and low end-tidal isoflurane concentration (FE’ISO). For bradycardia with hypotension, dogs received glycopyrrolate (0.005 mg/kg IV) or atropine (0.02 mg/kg IV). Hydromorphone (0.05 mg/kg IV) was used for hypertension or tachycardia. Postoperative pain management: Hydromorphone (0.05 mg/kg IV) was administered as rescue analgesia when dogs scored ≥ 6/24 on the Glasgow Composite Pain Scale (CMPS-SF). Statistical analysis used: Normality tested with Shapiro–Wilk test. Parametric data analysed using two-sample Student’s t-test. Nonparametric continuous data analysed using Wilcoxon rank sum test. Yes/no variables analysed using Fisher’s exact test.
Study design:	Prospective, blinded, randomised, controlled, clinical trial.
Outcome Studied:	Intraoperative assessments: Monitored every 15 minutes with heart rate (HR), mean arterial pressure (MAP), FE’ISO, and use of cardiovascular medications to evaluate cardiovascular performance and intraoperative pain. Postoperative pain: Assessed using CMPS-SF preoperatively (baseline), at extubation, and at 1, 4, and every 4 hours up to 24 hours post-operation. Recorded rescue analgesia administration postoperatively at relevant time points over a 24-hour period, as required based on specified pain scores. Postoperative residual block: Assessed by time to first urination, urinary retention, and ambulation (walking 10 metres on three or four limbs with an abdominal sling).
Main Findings (relevant to PICO question):	The F+S group had significantly higher average preoperative pain scores of 3 compared to 1 in the EPID group (P = 0.04). Intraoperative assessments: No significant differences in HR were observed between the F+S and EPID groups (F+S 76 ± 18 bpm; EPID 79 ± 18 bpm; P = 0.29). No significant differences in MAP (F+S 82 ± 8 mmHg; EPID 84 ± 8 mmHg; P = 0.40). No significant differences in anaesthetic requirements (FE′ISO: F+S 1.2 ± 0.2%; EPID 1.24 ± 0.2%, P = 0.38). No significant differences in the use of cardiovascular medications (phenylephrine: 3 dogs in each group, P = 1.00; atropine or glycopyrrolate: F+S 9 dogs; EPID 6 dogs, P = 0.43). Postoperative pain scores: Although the F+S group had a significantly higher baseline pain score (2) than the EPID group (1; P = 0.04), no significant differences were observed postoperatively (p-value not reported), with both groups peaking at 1 hour after extubation (EPID 5; F+S 4) and scores decreasing by 24 hours (EPID 1; F+S 2). Requirement for rescue analgesia: Within 30 minutes of extubation, 4/13 dogs in the EPID group and 3/13 dogs in the F+S group received rescue analgesia. Within 24 hours of extubation, 5/13 dogs in the EPID group and 4/13 dogs in the F+S group received rescue analgesia. No significant differences were found at either time point (P = 1.00 for both). Incidence of urinary retention: In the EPID group, dogs first urinated at 8 hours after extubation compared to 8.2 hours in the F+S group (p-value not reported). At 8 hours after extubation, urinary retention was observed in 3 dogs in the EPID group and 4 dogs in the F+S group, with no significant difference between groups (P = 1.00), and by 24 hours, no dogs in the EPID group and 1 dog in the F+S group had urinary retention (P = 1.00). Time to first ambulation: The EPID group took an average of 3.9 hours to first ambulate after extubation, while the F+S group took 3.5 hours, with no significant difference between the groups (P = 0.65). The EPID group took 9.2 hours, while the F+S group took 6.4 hours to ambulate after bupivacaine administration, with no significant difference between the groups (P = 0.27).
Limitations:	The lack of justification for the 2-point CMPS-SF increase as the basis for the sample size analysis, along with the absence of standard deviation data, limits confidence in the reliability and adequacy of the sample size. Variation of severity and duration of dogs' stifle lesions, as well as in prior treatments and medications such as pain relief and anti-inflammatory drugs. No specification regarding the experience levels of the veterinary surgeons. No specification regarding the surgical technique, using only the term 'stifle arthroplasty,' which limits the clarity and applicability of the findings. Physiological data during surgery were recorded at 15-minute intervals. Preexisting variability in preoperative pain scores between the F+S and EPID groups may have confounded the assessment of analgesic effectiveness. Although such differences can occur by chance in small randomised studies, the authors made no attempt to statistically control for this known confounder, which is particularly important given that pain was a primary outcome. The addition of dexmedetomidine in F+S and buprenorphine in EPID, along with medications such as hydromorphone, carprofen, and tramadol before and after surgery, introduced variability in assessing bupivacaine's analgesic effectiveness across both groups. Intraoperative pain was evaluated using physiological changes, which is not a validated approach. Three investigators were involved in the study, which introduces the possibility of inter-observer variation. No assessment of proprioceptive function, which is another important indicator of motor impairment. Exact p-values were sometimes not reported.

**Campoy et al. (2012):** Comparison of bupivacaine femoral and sciatic nerve block versus bupivacaine and morphine epidural for stifle surgery in dogs **Aim: **To determine whether a bupivacaine femoral and sciatic nerve block (F+S) provides superior analgesia, and fewer residual block effects compared to a bupivacaine and morphine epidural (EPID) in dogs undergoing unilateral tibial plateau levelling osteotomy (TPLO).

Population:	Healthy client-owned dogs with a median age of 3 (range 1–8 years), weighing 37 ± 11 kg, undergoing elective unilateral TPLO surgery.
Sample size:	20 dogs.
Intervention details:	Random allocation equally to one of two intervention groups: EPID group (n = 9, with 1 dog excluded due to failed EPID). Combined femoral and sciatic nerve block (F+S) group (n = 10). Anaesthesia and medication administration: All dogs received an NSAID (carprofen 2 mg/kg orally (PO) or 4 mg/kg subcutaneously (SC), deracoxib 2 mg/kg PO, or firocoxib 5 mg/kg PO) within 12 hours before anaesthesia. Premedication with acepromazine (0.02 mg/kg intramuscularly (IM) and hydromorphone (0.1 mg/kg IM) 30 minutes before anaesthesia induction with intravenous (IV) propofol, followed by maintenance with isoflurane in oxygen. Administration of respective treatment groups immediately before surgery: EPID: bupivacaine (0.5%, 0.5 mg/kg) + morphine (0.1%, 0.1 mg/kg in 0.2 mL/kg) F+S guided by electrolocation: bupivacaine (0.5%, 0.1 mL/kg) at each site. Intraoperative cardiovascular management: Dogs received dopamine infusion (5 μg/kg/minute IV) if they experienced hypotension despite a low end-tidal isoflurane concentration (FE'ISO) of 0.5%. Hydromorphone (0.05 mg/kg IV) was administered when hypertension or tachycardia persisted despite FE'ISO being 1.8%. Postoperative pain management: Hydromorphone (0.05 mg/kg IV) was given as rescue analgesia when dogs scored >5 on the Numerical Rating Scale (NRS). Statistical analysis used: Normality tested with Shapiro–Wilk test. Parametric data analysed with two-sample t-test. Non-parametric continuous data were analysed with the Wilcoxon rank-sum test, and categorical data with Fisher’s exact test.
Study design:	Prospective, blinded, randomised, controlled, clinical trial.
Outcome Studied:	Intraoperative assessments: Heart rate (HR), mean arterial pressure (MAP), FE’ISO, hypotension incidence, and dopamine use collected at 15-minute intervals to evaluate cardiovascular performance and intraoperative pain. Postoperative pain: Assessed using NRS with descriptions of their behaviour preoperatively (baseline), at extubation, and at 1, 4, and every 4 hours up to 24 hours postoperation. Recorded rescue analgesia requirements (administered based on specified pain scores) postoperatively at extubation and at intervals over a 24-hour period. Postoperative residual block: Assessed by time to first urination, urinary retention, and ambulation (walking 10 meters with an abdominal sling).
Main Findings (relevant to PICO question):	One dog in the EPID group was excluded from the study due to technique failure. Intraoperative assessments: EPID had a MAP of 74 ± 7, significantly lower than the F+S group with a MAP of 82 ± 11 (P = 0.04). EPID group had a significantly lower FE′ISO (0.9 ± 0.2) compared to the F+S group (1.1 ± 0.2) (P = 0.05). EPID had a lower HR (64 ± 14) compared to F+S (70 ± 11), but no significant difference was found (P = 0.26). The incidence of hypotension was similar in both groups, with 2 incidences each (P = 0.09). 6/9 dogs in the EPID group received dopamine, which was higher compared to the F+S group, where only 4/10 dogs received dopamine. However, this difference was not statistically significant (P = 0.24). Postoperative pain scores: Both groups had a baseline pain score of 2. At extubation, both groups scored 0, with pain scores increasing over time, reaching 4 in the EPID group and 3 in the F+S group by 24 hours postextubation; no significant differences were observed between the groups at any time point (p-value not reported). Requirement for rescue analgesia: At extubation, 2/9 dogs in the EPID group required hydromorphone, whereas none in the F+S group did (P = 0.21). In the F+S group, the first doses were administered to 2 dogs 4 hours later. 2/9 dogs in the EPID group and 3/10 dogs in the F+S group did not require hydromorphone, with no significant difference (P = 0.33). By 24 hours postanaesthesia recovery, the F+S group required significantly less overall use of rescue analgesia compared to the EPID group (0.05 mg/kg for F+S vs. 0.08 mg/kg for EPID; P = 0.04). Incidence of urinary retention: After surgery, 4/9 dogs in the EPID group developed urinary retention, whereas none in the F+S group were affected (P = 0.03). Time to first ambulation: The EPID group ambulated in about 8.3 hours postextubation, while the F+S group took approximately 8.7 hours postextubation, with no significant differences (P = 0.95). Both groups ambulated around 12 hours after the block, also showing no significant differences (P = 0.95).
Limitations:	Sample size was not calculated, posing a risk of type 2 error due to potentially small sample size. Lack of standardisation in severity and duration of original stifle joint lesions, as well as prior treatments with anti-inflammatory and analgesic drugs. Lack of specification regarding the veterinary surgeons' experience, the surgical approach to stifle joint inspection, or whether joint inspection was performed, potentially confounding the study results. The use of electrolocation for F+S, which is considered less precise and associated with greater tissue damage and longer procedure times compared to ultrasound. NSAIDs and premedications, known for their analgesic and sedative effects, alongside the addition of morphine to bupivacaine in EPID, may have influenced both the analgesic outcomes and the incidence of residual blocks, potentially confounding the study results. Use of a non-validated physiological tool for assessing intraoperative pain. Intraoperative data recorded every 15 minutes might have led to missed significant findings in pain assessment. Did not assess proprioceptive function, which is another important indicator of motor impairment. Incomplete reporting of exact p-values in some instances.

**Caniglia et al. (2012):** Intraoperative antinociception and postoperative analgesia following epidural anesthesia versus femoral and sciatic nerve blockade in dogs undergoing stifle joint surgery **Aim: **To compare the analgesic efficacy of bupivacaine and lignocaine femoral and sciatic nerve block (F+S) versus bupivacaine and lignocaine epidural (EPID) during the pre-, intra-, and postoperative periods in dogs undergoing surgery for cranial cruciate ligament disease (CrCL) and medial patellar luxation (MPL).

Population:	Healthy client-owned dogs in the United States, ranging in age from 2 to 12 years and weighing between 11.2 and 60.3 kg, undergoing surgical procedures for CrC or MPL.
Sample size:	22 dogs.
Intervention details:	Random allocation equally to one of two intervention groups: EPID group (n = 11) F+S group (n = 10, with one dog excluded due to a blinding procedure failure) Anaesthesia and medication administration: Premedication with acepromazine (0.02 mg/kg intramuscularly (IM)) and morphine (0.3 mg/kg IM) before anaesthesia induction with diazepam (0.3 mg/kg intravenously (IV)) and propofol (2 to 6 mg/kg IV), followed by maintenance with sevoflurane in oxygen. Administration of treatments before surgery: EPID: lignocaine 1.0% (2 mg/kg) and bupivacaine 0.25% (0.5 mg/kg), given at 0.2 mL/kg, up to a total of 6 mL. F+S guided by electrolocation: lignocaine 1.0% solution and bupivacaine 0.25% solution at 0.1 mg/kg for femoral nerve and 0.3 mg/kg for sciatic nerve. Intraoperative cardiovascular management: Dogs received fentanyl (2 μg/kg IV) if heart rate (HR) or systolic arterial pressure (SAP) increased > 10% from baseline during surgery with adequate anaesthetic depth, to restore values to baseline. Hypotensive episodes were initially managed with an IV bolus of crystalloid solution (10 mL/kg). If hypotension persisted, dogs received either glycopyrrolate (10 μg/kg) or dopamine (5 to 20 μg/kg/min). Postoperative pain and anxiety management: Hydromorphone (0.05 mg/kg IV) was administered as rescue analgesia when dogs scored > 5/20 on the Glasgow Composite Pain Scale (CMPS-SF). Acepromazine (5 μg/kg IV) was administered to manage anxiety or central nervous system (CNS) excitation. Dogs unresponsive to acepromazine received hydromorphone (0.1 mg/kg IV). Statistical analysis used: Homogeneity analysis using the Fisher exact test to explore patient and clinical management distribution. Comparison of interval variables using Kruskal–Wallis tests to examine age, body weight, and other factors across groups. Intraoperative measures compared using a random intercept regression model to analyse HR, SAP, End-tidal sevoflurane concentration (FE’SEVO), and End-tidal carbon dioxide (ET CO_2_), with analysis of variance (ANOVA) used specifically for ETCO_2_. The timing of first postoperative analgesia was analysed with Kaplan–Meier survival and Cox regression. Pain score analysis transformed scores using Tukey's method and the Shapiro–Francia test for normalisation, followed by two-sample t-tests.
Study design:	Prospective, blinded, randomised, controlled, clinical trial.
Outcome Studied:	Intraoperative assessments: Monitored HR, SAP, FE’SEVO every 5 minutes during anaesthesia and recorded cardiovascular treatments. Compared EPID and F+S groups at key surgery phases. Postoperative pain assessment: Assessed using CMPS-SF immediately after extubation (baseline) and every 30 minutes for 360 minutes. Recorded rescue analgesia requirements (administered at specified pain scores) during the 360-minute postoperative observation period.
Main Findings (relevant to PICO question):	One dog in the F+S group experienced sciatic nerve deficits for 18 hours after the block but resolved 30 hours after the block. Intraoperative pain: Both groups had similar HR (EPID: 106 ± 22; F+S: 101 ± 22), SAP (EPID: 93 ± 23; F+S: 93 ± 15), and FE’SEVO (EPID: 1.94 ± 0.07; F+S: 97 ± 0.11), with no significant differences at any time point (p-values not reported). Postoperative pain scores: No significant difference in baseline scores (EPID: 0.44 ± 0.35; F+S: 0.57 ± 0.89; P = 0.67), or in scores at any time point from 30 to 360 minutes (exact values and p-values not reported). Requirement for rescue analgesia: Intraoperatively, 3/11 dogs in the EPID group required 1 fentanyl bolus and 2/11 required 2 boluses, compared with 3/9 dogs in the F+S group requiring 1 bolus. No significant difference was identified between groups (P = 0.55). The first postoperative rescue was given at 342.0 ± 45.0 minutes for EPID and 325.0 ± 68.0 minutes for F+S, with no significant difference (P = 0.49). 2/11 dogs in the EPID group and 4/9 dogs in the F+S group received hydromorphone within 360 minutes, but this difference was not statistically significant (P = 0.51).
Limitations:	No sample size calculation or justification was provided. Lack of standardisation regarding the surgical techniques (tibial-plateau levelling osteotomy (TPLO), lateral suture stabilisation, and commercial cruciate ligament repair systems) may have confounded the results, as these procedures differ in both intraoperative and postoperative pain profiles. Lack of control over the nature, severity, and duration of the original stifle joint lesion, as well as prior anti-inflammatory and analgesic drug treatments. The use of electrolocation for F+S, which is considered less precise and associated with greater tissue damage and longer procedure times compared to ultrasound. The electrolocation technique employed for approximating nerves is not consistent with conventional approaches. Variable volumes and mixed anaesthetics (lignocaine and bupivacaine) used for femoral and sciatic nerves could result in variability in block effectiveness. Personnel qualifications for performing the nerve blocks were unspecified. Non-validated physiological tools were used for intraoperative assessments. Use of medications like acepromazine and morphine with known sedative or analgesic effects complicates assessment of bupivacaine's analgesic efficacy. No preoperative CMPS-SF pain scores were collected, potentially confounding results due to residual anaesthetic effects and surgical pain. Exact p-values were not reported for all data points.

**Graff et al. (2024):** A comparison of the motor effects and analgesic efficacy following lumbar plexus block combined with sciatic nerve block or epidural in dogs undergoing tibial plateau leveling osteotomy **Aim: **To compare analgesic efficacy and the incidence of residual block between lumbar plexus and sciatic nerve block (LPSNB) using bupivacaine, and epidural anaesthesia (EPID) using morphine and bupivacaine in dogs undergoing tibial plateau levelling osteotomy (TPLO).

Population:	Healthy, client-owned dogs of medium to large breeds in the United States, ages ranging from 5.2 to 8.5 years (mean 7 years) in the EPID group and ages ranging from 3.5 to 8.2 years (mean 6.2 years) in the LPS group, weighing at least 20 kg, undergoing TPLO surgery.
Sample size:	30 dogs.
Intervention details:	Random allocation to one of two intervention groups: EPID group (n = 12, with one withdrawn due to epidural injection failure). LPS group (n = 15, with one withdrawn due to propofol and hydromorphone administration). One excluded due to rescheduling. Anaesthesia and medication administration: Premedication with acepromazine (0.02 mg/kg intramuscularly (IM)) and hydromorphone (0.1 mg/kg IM) prior to induction of anaesthesia with intravenous (IV) propofol and maintained with isoflurane in oxygen. At the end of surgery, all dogs were administered carprofen (4 mg/kg, 50 mg/mL subcutaneously (SC)). Administration of treatments before surgery: EPID: a combination of morphine (0.1 mg/kg, Duramorph 0.1%) and bupivacaine (0.5 mg/kg, 0.75% Marcaine) with a final volume of 0.167 mL/kg. Lumbar plexus guided by ultrasound: bupivacaine (1.3 mg/kg; 7.5 mg/mL). Sciatic nerve guided by ultrasound: bupivacaine (0.7 mg/kg; 7.5 mg/mL). Intraoperative cardiovascular assessment: Dogs received propofol (1 mg/kg IV) if their heart rate (HR) or respiratory rate (RR) increased by more than 20% or if they showed spontaneous movement after towel clamping, skin incision, joint incision, or the start of skin suturing. A second dose of propofol was administered if HR or RR increased by more than 20% following another stimulus. A third increase in HR or RR to another stimulus-prompted administration of hydromorphone (0.05 mg/kg IV). Postoperative pain management: Dogs with a Glasgow Composite Pain Scale (CMPS-SF) score of 6/24 or 5/20 were administered hydromorphone (0.05 mg/kg IV or IM)). Statistical analysis used: Comparison of continuous variables between LPS and EPID groups: Student’s t-test was used for normally distributed data. Wilcoxon rank sum test was used for non-normally distributed data. Fisher’s exact test was used to analyse categorical variables.
Study design:	Prospective, blinded, randomised, controlled, clinical trial.
Outcome Studied:	Intraoperative assessment: Monitored physiological parameters (HR and RR). Recorded intraoperative rescue analgesia (propofol and hydromorphone) based on changes in HR or RR. Postoperative pain assessment: Assessed with CMPS-SF before premedication (baseline) and at relevant timepoints over a 24-hour period. Recorded postoperative rescue analgesia requirement (hydromorphone) based on specified pain levels. Postoperative residual block: Assessed by time to first spontaneous urination, time to stand and walk using abdominal sling support and verbal commands, and immediate pelvic limb motor function score following each pain assessment.
Main Findings (relevant to PICO question):	One dog in the EPID group encountered a failure in EPID administration. One dog in the LPS group was withdrawn due to propofol and hydromorphone administration. Intraoperative pain: 3 dogs in the EPID group and 1 in the LPS group received propofol intraoperatively due to HR exceeding the predetermined threshold, but this difference was not statistically significant (P = 0.438). Postoperative pain scores: No significant differences were observed at any time points. At extubation, EPID scored 1 and LPS scored 2 (P = 0.122). EPID peaked at 3.7 at 120 minutes post-extubation, while LPS scored 3 (P = 0.603). LPS reached a peak of 3.7 at 240 minutes, while EPID scored 2 (P = 0.782). At 24 hours post-extubation, both groups scored 1 (P = 0.822). Time to spontaneous urination: Both groups had similar times to spontaneous urination. EPID took 300 minutes and LPS took 330 minutes after extubation, with no significant difference (P = 1.00). Time to first ambulation: Dogs in the LPS group regained mobility more quickly than those in the EPID group, standing at approximately 60 minutes compared with 150 minutes after extubation (P = 0.003), and walking at 90 minutes compared with 180 minutes, respectively (P = 0.006). At 1 hour after extubation, LPS dogs had a better overall motor score of 3, compared to 5 in the EPID group (P = 0.014). However, motor function was similar between groups at most postoperative evaluation times, with both groups scoring similarly from 5 at extubation (P = 0.872), gradually decreasing to 1 by 24 hours after extubation (P = 0.369), with no significant differences observed at the remaining time points (P > 0.05).
Limitations:	The study used a weak basis for sample size calculation, using pilot data from only three dogs with wide variability, likely underestimating sample size; power for other pain outcomes was also not calculated, leaving study power unclear and increasing risk of type 2 error. The severity, duration of stifle joint lesions, and prior treatments varied. The study showed enrolment bias by only including medium to large breed dogs. The study used an LPS approach instead of combined femoral and sciatic nerve block (F+S), complicating comparisons with other studies. All dogs received a non-steroidal anti-inflammatory drug (NSAID) post-surgery, potentially affecting bupivacaine's analgesic assessment. Morphine combined with bupivacaine in EPID introduced variability in pain relief and residual block incidences. Uneven volumes used in lumbar plexus and sciatic nerve blocks. Did not assess proprioceptive function, which is another important indicator of motor impairment. Exact p-values were not always reported.

**Kalamaras et al. (2021):** Effects of perioperative saphenous and sciatic nerve blocks, lumbosacral epidural or morphine–lidocaine–ketamine infusion on postoperative pain and sedation in dogs undergoing tibial plateau leveling osteotomy **Aim: **To compare postoperative analgesic efficacy and the incidence of residual block between morphine–lidocaine–ketamine constant rate infusion (MLK CRI), epidural anaesthesia (EPID), and saphenous and sciatic nerve block (SSNB) in dogs undergoing elective unilateral tibial plateau levelling osteotomy (TPLO).

Population:	Healthy client-owned dogs, ages ranging from 1 to 12 years (mean 5.2 years), weight ranging from 15.9 to 56.7 kg (mean 33.9 kg), undergoing elective unilateral TPLO surgery.
Sample size:	30 dogs.
Intervention details:	Statistical analysis used: Morphine (0.4 mg/kg) was administered IM as rescue analgesia to any dog scoring > 5 on the Glasgow Composite Pain Scale (CMPS-SF). Postoperative pain management: EPID: ropivacaine (1%, 0.2 mg/kg) and morphine (0.05%, 0.09 mg/kg) in a total volume of 0.2 mL/kg SSNB guided by ultrasound and electrolocation: ropivacaine (1%, 1mg/kg) at each site. Administration of treatments 30–60 minutes before surgery: Premedication with acepromazine (0.05 mg/kg intramuscularly (IM) and morphine (0.2 mg/kg IM) before anaesthesia induction with intravenous (IV) propofol, followed by maintenance with isoflurane in oxygen. Postextubation, all dogs received a non-steroidal anti-inflammatory drug (NSAID): carprofen (2.2 mg/kg subcutaneously (SC) or meloxicam (0.1 mg/kg SC) or deracoxib (2 mg/kg orally (PO)). Gabapentin(10–20 mg/kg PO) and trazodone (4–10 mg/kg PO) were given every 8–12 hours, starting with the first meal after surgery. Anaesthesia and medication administration: Morphine–lidocaine–ketamine continuous rate infusion (MLK CRI) group (n = 15). The results of the MLK CRI group do not relate to the PICO question and therefore will not be commented on further in this Knowledge Summary. Epidural anaesthesia (EPID) group (n = 15). Saphenous and sciatic nerve block (SSNB) group (n =15). Random allocation equally to one of three intervention groups: Demographic data were compared between treatment groups using analysis of variance (ANOVA). Linear mixed-effects models were used to assess the effect of treatment group on primary outcomes. Group means were compared at each postoperative time point using preplanned contrasts. Kenward–Roger adjustment was used to control type 1error because outcomes were moderately non-normally distributed.
Study design:	Prospective, blinded, randomised, controlled, clinical trial.
Outcome Studied:	Pain was evaluated using CMPS-SF and Colorado State University canine acute pain scale (CSU-CAPS) immediately after extubation and at 2, 4, 8, and 24 hours thereafter. In case of discrepancies between the CMPS-SF and CSU-CAPS scores, the CMPS-SF total score was used to determine the need for rescue analgesia. Rescue analgesia administration was recorded, and the dog was reevaluated using both CMPS-SF and CSU-CAPS after 60 minutes.
Main Findings (relevant to PICO question):	Postoperative pain scores: At 4 and 8 hours postoperatively, the SSNB group had significantly lower pain scores than the EPID group, with SSNB scoring 3 and EPID scoring 4 for both CSU-CAPS (P = 0.0017) and CMPS-SF (P = 0.0007) systems. No significant difference was observed 2 hours postoperatively, with SSNB and EPID groups scoring 3 and 4 respectively, and both groups scoring 3 at 24 hours postoperatively (p-values were not reported for either time point). Requirement for rescue analgesia: No dogs required rescue analgesia within 24 hours postoperatively. Postoperative residual block: Although not specifically studied, all dogs urinated within 24 hours after surgery and were able to walk before being discharged the next day.
Limitations:	Sample size was based on a previous study involving the same dog population, and power was not calculated for the current study variables, increasing the risk of type 2 error due to a potentially small sample size. Enrolment biased towards medium-to-large breed dogs. Use of ropivacaine complicates comparison with studies using bupivacaine. Use of SSNB approach complicates comparison with combined femoral and sciatic nerve block (F+S) or lumbar plexus and sciatic nerve block (LPS) techniques. Lack of standardisation in severity and duration of original stifle joint lesions, as well as prior treatments with anti-inflammatory and analgesic drugs. Inherent differences among the three NSAIDs were not controlled, potentially confounding results. NSAIDs and premedications, known for their analgesic and sedative effects, alongside the addition of morphine to ropivacaine in EPID, may have influenced the analgesic outcomes, potentially confounding the study results. Use of two assessors for pain scoring may have introduced interobserver variation. The CSU-CAPS has not been validated for assessing postoperative pain in dogs.

**McCally et al. (2015):** Comparison of Short-Term Postoperative Analgesia by Epidural, Femoral Nerve Block, or Combination Femoral and Sciatic Nerve Block in Dogs Undergoing Tibial Plateau Leveling Osteotomy **Aim: **To compare the short-term postoperative analgesic efficacy of bupivacaine epidural (EPID), bupivacaine femoral and sciatic nerve block (F+S), and bupivacaine femoral nerve block (FNB) in dogs undergoing tibial plateau levelling osteotomy (TPLO).

Population:	Healthy client-owned dogs, ages ranging from 1 to 8 years (mean 4.3 years) and weighing between 20 kg to 58 kg, undergoing TPLO surgery.
Sample size:	45 dogs.
Intervention details:	Random allocation to one of three intervention groups: EPID group (n = 14) F+S group (n = 17) FNB group (n = 14). The results of the femoral nerve block do not relate to the PICO question and therefore will not be commented on further in this Knowledge Summary. A sample size calculation with 80% power and a significance level of 0.05 determined that 14 dogs per group were needed. Anaesthesia and medication administration: Premedication with dexmedetomidine (5 μg/kg intramuscularly (IM)) + hydromorphone (0.1 mg/kg IM)) prior to induction of anaesthesia with intravenous (IV) propofol, then maintained with isoflurane in oxygen. Administration of treatments before surgery: EPID: 0.5% bupivacaine at 0.2 mL/kg, maximum 6 mL/dog. F+S guided by electrolocation: 0.5% bupivacaine with 0.2 mL/kg administered at each site. Postoperative pain management: For dogs scoring ≥ 6 overall or ≥ 3 in any Glasgow Composite Pain Scale (CMPS-SF) category, hydromorphone (0.05 mg/kg IV) was administered. If pain scores remained ≥ 6 upon reassessment, Dexmedetomidine (2 μg/kg IV) was given for further analgesia. Statistical analysis: CMPS-SF scores within each treatment were compared using Friedman repeated measures analysis of variance (ANOVA) on ranks. Kruskal–Wallis tests compared CMPS-SF scores across treatments and time to first rescue. Post hoc Wilcoxon rank sum tests were used for pairwise comparisons of significant differences in CMPS-SF scores between treatments. The proportions of dogs requiring rescue analgesia across treatments were compared using Fisher's exact test. Anaesthesia and surgery durations were using Shapiro–Wilk tests for normality and compared across treatments using Student's t-tests. Body weight comparisons across treatments were conducted using a one-way ANOVA, while age comparisons were performed using a Kruskal–Wallis 1-way ANOVA.
Study design:	Prospective, blinded, randomised, controlled, clinical trial.
Outcome Studied:	CMPS-SF scores at extubation (baseline) and at 1, 2, 4, 6, and 8 hours after extubation. Rescue analgesia requirement (administered at specified pain levels) during the 8-hour postoperative period.
Main Findings (relevant to PICO question):	Postoperative pain scores: At extubation, the FNB group had a significantly higher CMPS-SF score of 3 compared to F+S, which scored 2 (P = 0.033). The EPID group also scored 2, but the difference from FNB was not statistically significant (p-value not reported). No significant differences were observed at any other time points (p-values not reported). At 8 hours post-extubation, FNB had a score of 1.5, F+S scored 1, and EPID scored 2. Requirement for rescue analgesia: 4/14 dogs in the FNB group required rescue analgesia at extubation, which was significantly higher than in the F+S group, where none of the seventeen dogs required it (P = 0.037). Although 2/14 dogs in the EPID group required rescue analgesia at extubation, this was not significantly different from FNB (P = 0.39), nor was there a significant difference between F+S and EPID (P = 0.18). No significant difference in the proportion of dogs requiring at least one rescue analgesia (P > 0.14, exact values not reported) or two or more doses (P > 0.56, exact values not reported). There was no significant difference in the time to first rescue between FNB (0 hours), F+S (2 hours), and EPID (1.5 hours) (p-values not reported).
Limitations:	The severity, duration, and treatment history of the original stifle joint lesion was not standardised. Study enrolment biased towards medium to large breed dogs. The lack of justification for the 2-point CMPS-SF difference as the basis for the sample size analysis, along with the absence of standard deviation data, limits confidence in the reliability and adequacy of the sample size. Lack of specification regarding the number and doses of rescue analgesia used at specific time points complicates further analysis. No preoperative CMPS-SF pain scores were collected, potentially confounding results due to residual anaesthetic effects and surgical pain. The use of electrolocation for F+S, which is considered less precise and associated with greater tissue damage and longer procedure times compared to ultrasound. Three observers were involved in the assessments, which introduces the possibility of inter-observer variation. Exact p-values were occasionally not reported.

## Appraisal, application and reflection

Cranial cruciate ligament disease (CrCL) and medial patella luxation (MPL) are common stifle conditions in dogs that frequently cause substantial pain, often necessitating surgical management (Caniglia et al., 2012). Patients undergoing these surgeries frequently experience significant perioperative pain (Bartel et al., 2016). Regional anaesthesia techniques with bupivacaine or ropivacaine are commonly used to manage this pain (Thomson et al., 2021; Kalamaras et al., 2021), with various administration methods available. One method is epidural anaesthesia (EPID), where bupivacaine or ropivacaine is injected into the epidural space to block pain signals to the spinal cord (Valverde, 2008). EPID has an 85% success rate (Sarotti et al., 2022), but it can cause postoperative urinary retention for 24 hours or longer in approximately 3.5% of cases (Campoy et al., 2012; Bartel et al., 2016). As an alternative, locoregional nerve blocks, particularly those using combined nerve blocks such as combined femoral and sciatic nerve block (F+S), saphenous and sciatic nerve block (SSNB), and lumbar plexus and sciatic nerve block (LPS), identified via electrolocation or ultrasound, have gained popularity among veterinary surgeons (Thomson et al., 2021). These techniques offer a lower incidence of residual motor block while maintaining a success rate similar to that of EPID (Graff et al., 2024; Vettorato et al., 2012). While there is a risk of neurological deficits, it is minimal at only 1.05%, and the effects are transient (Vettorato et al., 2012).

To address the PICO, six prospective, blinded, randomised, controlled, clinical trials were reviewed, with no observational studies included. Three studies (Campoy et al., 2012; Caniglia et al., 2012; Bartel et al., 2016) compared F+S and EPID. McCally et al. (2015) compared femoral nerve block (FNB), F+S, and EPID, Kalamaras et al. (2021) compared SSNB and EPID, and Graff et al. (2024) compared LPS with EPID. Of the studies appraised, all used client-owned dogs and excluded those with pre-existing diseases. However, McCally et al. (2015), Kalamaras et al. (2021), and Graff et al. (2024) limited their study populations to medium and large breed dogs, excluding small breeds under 10 kg, an important subgroup also predisposed to stifle lesions (Olimpo et al., 2016). Additionally, Campoy et al. (2012) and Caniglia et al. (2012) did not perform sample size calculations, which may have reduced their ability to detect differences between techniques. Although McCally et al. (2015) and Bartel et al. (2016) performed sample size calculations, the lack of justification for using a 2-point Glasgow Composite Pain Scale (CMPS-SF) difference and the absence of standard deviation data reduce confidence in their adequacy. Kalamaras et al. (2021) based their sample size calculation on a previous study involving the same dog population, while Graff et al. (2024) relied on pilot data from only three dogs; moreover, power was not calculated for current study variables, leaving the study’s overall power unclear and increasing the risk of type 2 error. Furthermore, inconsistent p-value reporting complicates interpretation. While none of the studies exhibited major methodological flaws that would invalidate their findings, the results should be interpreted with caution, particularly regarding the strength and reliability of the conclusions drawn.

Taking these limitations into account, the studies will be analysed to identify the optimal technique with the highest success rates, maximal analgesic efficacy, and minimal residual block incidences. In terms of success rates, Campoy et al. (2012) and Graff et al. (2024) each reported a single failure with EPID. Meanwhile, all F+S, SSNB, and LPS approaches, guided by either ultrasound or electrolocation, achieved 100% success rates in all studies, with only one case of transient neurological deficit (Caniglia et al., 2012). This supports findings of the comparable success rates between EPID and locoregional nerve blocks (Sarotti et al., 2022; Vettorato et al., 2012), as well as a low risk of neurological complications (Vettorato et al., 2012). As success rates for both methods are comparable and high, analgesic efficacy and incidence of residual block will be the remaining criteria used to determine the preferred technique.

When evaluating the analgesic efficacy of EPID and F+S techniques, Campoy et al. (2012) suggested that EPID might offer superior intraoperative analgesia, as indicated by lower mean arterial pressure (MAP) and end-tidal isoflurane concentration (FE'ISO) in the EPID group. However, this conclusion was not consistently supported by other studies. Caniglia et al. (2012), Bartel et al. (2016), and Graff et al. (2024) found no significant differences in intraoperative pain between techniques, while McCally et al. (2015) and Kalamaras et al. (2021) did not assess intraoperative pain. Postoperatively, McCally et al. (2015) found that FNB alone resulted in significantly higher CMPS-SF scores at extubation compared to F+S, and more dogs in the FNB group required rescue analgesia at extubation. These findings suggest that FNB alone may be insufficient and that a combined approach, typically a F+S, SSNB or LPS, is needed for effective analgesia. When comparing combined locoregional nerve blocks to EPID, Campoy et al. (2012) found that the EPID group required a higher overall dose of hydromorphone for analgesia compared to F+S, despite similar pain scores. Kalamaras et al. (2021) found that the SSNB group had significantly lower pain scores than the EPID group at 4 and 8 hours postoperatively for both Colorado State University canine acute pain scale (CSU-CAPS) and CMPS-SF systems, with no significant differences at other time points. In contrast, the studies by Caniglia et al. (2012), McCally et al. (2015), Bartel et al. (2016), and Graff et al. (2024) found no differences in pain scores or rescue analgesia requirements between EPID and F+S or LPS. These inconsistencies between studies may be attributed to methodological limitations, which are discussed below.

One major issue is the lack of standardisation in study methodologies when assessing analgesic efficacy, which introduces several potential confounding factors. For instance, Bartel et al. (2016) reported higher baseline pain scores in the F+S group despite randomisation and did not statistically control for this known confounder. Given that analgesic efficacy was a primary outcome, this reduces the internal validity of the study. Variability in stifle joint lesion severity, duration, and prior treatments further complicates comparisons. Additionally, the unreported experience levels of surgeons in Campoy et al. (2012) and Bartel et al. (2016) impact procedural consistency. The lack of specification of surgical techniques in both Campoy et al. (2012) and Bartel et al. (2016), along with the absence of standardisation of surgical techniques in Caniglia et al. (2012), could have confounded results, as these surgical techniques vary in both intraoperative and postoperative pain profiles, which are key study outcomes. Furthermore, the use of premedications and pain relief, while reflective of real veterinary practice, introduces additional sedation or analgesic effects that complicate the assessment of the local anaesthesia techniques used. Variation in drug protocols across studies further complicates interpretation. Together, these factors collectively weaken the quality of the evidence. Therefore, further research is required to clarify the comparative analgesic efficacy of locoregional nerve blocks and EPID, using standardised methodologies to enhance the validity of future findings.

Moreover, specific methodological limitations in various studies may have masked the potential analgesic differences between locoregional nerve blocks and EPID technique. For example, Bartel et al. (2016) collected data every 15 minutes instead of the recommended 5- to 10-minute intervals (Bednarski et al., 2011), which might have resulted in missing significant findings. Although Campoy et al. (2012) also used 15-minute intervals and reported significant results, similar limitations could have obscured more substantial findings. In administering F+S bupivacaine, three studies (Campoy et al., 2012; Caniglia et al., 2012; and McCally et al., 2015) used electrolocation, which is less precise than ultrasound (Campoy et al., 2010), potentially confounding the results. Furthermore, Caniglia et al. (2012) used 0.3 mA as the minimum current for nerve localisation instead of the standard 0.2 mA (Mahler & Adogwa, 2008), which might have placed the needle too far from the nerve and reduced block efficacy. The use of a lignocaine-bupivacaine mix in Caniglia et al. (2012) and uneven bupivacaine volumes in both Caniglia et al. (2012) and Graff et al. (2024) could have influenced the results. Overall, these methodological limitations introduce uncertainties that reduce the reliability of the conclusions drawn.

Finally, the inherent complexity of pain presents significant challenges, leading to a high potential for errors in current veterinary pain measures. Although studies used objective physiological measures to assess intraoperative pain, these measures are not validated and can be influenced by non-pain factors (Hernandez-Avalos et al., 2019). Additionally, pain scoring systems used to assess postoperative pain, such as the Numerical Rating Scale (NRS), are subjective and may exhibit high inter-observer variability (Hernandez-Avalos et al., 2019). Furthermore, the CSU-CAPS used in the study by Kalamaras et al. (2021) has not been validated for postoperative pain assessment in dogs. Therefore, despite evidence suggesting similar pain control between techniques, methodological issues make definitive conclusions challenging.

The last criterion evaluated is residual block, with urinary retention and ambulation assessed in three of the six studies (Campoy et al., 2012; Bartel et al., 2016; Graff et al., 2024). These studies monitored urinary retention lasting more than 24 hours following standard postoperative protocols. While Bartel et al. (2016) and Graff et al. (2024) found no significant differences in urinary retention, Campoy et al. (2012) reported that 44% (4/9) of dogs in the EPID group experienced urinary retention (P = 0.03), possibly linked to morphine (Herperger, 1998). However, more recent studies by Peterson et al. (2014) and Graff et al. (2024) have not definitively linked EPID morphine to urinary retention. This leaves open the possibility that epidural bupivacaine administration alone could contribute to this issue, indicating that further research is needed.

In terms of ambulation, Graff et al. (2024) found that dogs in the LPS group took less time to stand (P = 0.003) and walk (P = 0.006), with better motor function at 1 hour post-extubation (P = 0.014). Campoy et al. (2012) and Bartel et al. (2016) did not find significant results, likely due to methodological differences. Specifically, Graff et al. (2024) used a higher bupivacaine dosage and employed a LPS approach compared to the F+S technique used by Campoy et al. (2012) and Bartel et al. (2016), which may explain the differences in findings. However, the reliability of these findings may be compromised by the incomplete assessment of motor function across all three studies. Campoy et al. (2012) and Bartel et al. (2016) focused only on the time to first ambulation, whereas Graff et al. (2024) provided a more comprehensive evaluation by assessing pelvic limb motor scores in addition to the time taken to stand and walk. Nevertheless, these assessments are limited because dogs can walk on three legs, potentially leading to a false impression that they have fully regained motor function. Therefore, conducting proprioceptive assessments of all four limbs is essential for a more accurate evaluation of motor function. Overall, although evidence suggests that locoregional nerve blocks may reduce residual block, the results are inconsistent, and certain methodological limitations diminish the quality of the findings.

In conclusion, the literature provides moderate evidence that locoregional nerve blocks and EPID are comparable techniques for intraoperative and postoperative analgesia. There is low to moderate evidence that locoregional nerve blocks may have advantages over EPID for reducing residual block. Further research is needed to enhance the clinical applicability of locoregional nerve blocks compared to epidural analgesia for pain management in dogs undergoing stifle surgery.

## Methodology

**Search Strategy:** 

Databases searched and dates covered:	CAB Abstracts on OVID platform 1973–Week 16 2025 PubMed on University of Sydney Library search platform (1946–April 2025) Web of Science on University of Sydney Library search platform (1910 –April 2025)
Search strategy:	CAB Abstracts: (dog* or canis or canine or canid*).mp (stifle or knee* or joint* or limb* or hind limb or hind-limb or hindlimb or pelvic limb or patella* or orthopaedic* or tibial plateau levelling osteotomy or TPLO or medial patella luxation or MPL).mp (bupivacaine or ropivacaine).mp (epidural* or extradural* or extra-dural* or extrathecal* or extra-thecal*).mp ("peripheral nerve block*" or "peripheral nerve blockade" or "femoral and sciatic nerve block*" or "femoral and sciatic nerve blockade" or "combination femoral and sciatic nerve block*" or “saphenous and sciatic nerve block” or “saphenous and sciatic nerve blocks” or “locoregional nerve block” or “locoregional nerve blocks” or “locoregional nerve blockade”).mp (pain or perioperative analgesia or perioperative antinociception or intraoperative analgesia or intraoperative antinociception or analgesia or postoperative analgesia or short-term postoperative analgesia or postoperative pain score or pain score or pain scale).mp 1 and 2 and 3 and 4 and 5 and 6 PubMed:(dog OR dogs OR canis OR canine OR canid*) AND (stifle OR knee* OR joint OR joints OR limb OR limbs OR “hind limb” OR hind-limb OR hindlimb OR pelvic limb OR patella* OR orthopaedic* OR orthopaedic* OR tibial plateau levelling osteotomy OR TPLO OR MPL OR MPL) AND (bupivacaine OR ropivacaine) AND (epidural* OR extradural* OR “extra dural*” OR extra-dural* OR extrathecal* OR “extra thecal*” OR extra-thecal*) AND (peripheral nerve block OR peripheral nerve blockade OR femoral and sciatic nerve block OR femoral and sciatic nerve blockade OR combination femoral and sciatic nerve block OR saphenous and sciatic nerve block OR saphenous and sciatic nerve blocks OR locoregional nerve block OR locoregional nerve blocks OR locoregional nerve blockade) AND (pain OR perioperative analgesia OR perioperative antinociception OR intraoperative analgesia OR intraoperative antinociception OR analgesia OR postoperative analgesia OR short-term postoperative analgesia OR postoperative pain score OR pain score OR pain scale)Web of Science:(TS=(dog OR dogs OR canis OR canine OR canid*) AND TS=(stifle OR knee* OR joint OR joints OR limb OR limbs OR “hind limb” OR hind-limb OR hindlimb OR pelvic limb OR patella* OR orthopaedic* OR orthopaedic* OR "tibial plateau levelling osteotomy" OR TPLO OR "MPL" OR MPL) AND TS=(bupivacaine OR ropivacaine) AND TS=(epidural* OR extradural* OR "extra dural*" OR extra-dural* OR extrathecal* OR "extra thecal*" OR extra-thecal*) AND TS=(“peripheral nerve block” OR "peripheral nerve blockade" OR “femoral and sciatic nerve block” OR "femoral and sciatic nerve blockade" OR “combination femoral and sciatic nerve block” OR “saphenous and sciatic nerve block” OR “saphenous and sciatic nerve blocks” OR “locoregional nerve block” OR “locoregional nerve blocks” OR “locoregional nerve blockade”) AND TS=(pain OR “perioperative analgesia” OR “perioperative antinociception” OR “intraoperative analgesia” OR “intraoperative antinociception” OR analgesia OR “post-operative pain score” OR “pain score” OR “pain scale”)
Dates searches performed:	18 April 2025

**Exclusion / Inclusion Criteria:** 

Exclusion:	Non peer-reviewed studies. Pilot studies and non primary studies. Studies not available in English. Studies that did not address the PICO question.
Inclusion:	Studies with interventions that consisted of locoregional nerve blocks and EPID bupivacaine or ropivacaine administration in dogs undergoing stifle surgeries under general anaesthesia.

**Search Outcome:** 

Database	Number of results	Excluded – not peer reviewed	Excluded – pilot studies and non primary studies	Excluded – non-English publication	Excluded – not relevant to the PICO	Total relevant papers
CAB Abstracts	10	0	1	1	3	5
PubMed	15	0	4	1	4	6
Web of Science	12	0	2	1	5	4
Total relevant papers when duplicates removed						6

## ORCiD

Tiffany Tang: https://orcid.org/0009-0008-7312-0544

Eduardo Uquillas: https://orcid.org/0000-0002-4227-2173

## Conflict of Interest

The authors declare no conflicts of interest.

## Disclaimer

Knowledge Summaries are a peer-reviewed article type which aims to answer a clinical question based on the best available current evidence. It does not override the responsibility of the practitioner. Informed decisions should be made by considering such factors as individual clinical expertise and judgement along with patient’s circumstances and owners’ values. Knowledge Summaries are a resource to help inform and any opinions expressed within the Knowledge Summaries are the author's own and do not necessarily reflect the view of the RCVS Knowledge. Authors are responsible for the accuracy of the content. While the Editor and Publisher believe that all content herein are in accord with current recommendations and practice at the time of publication, they accept no legal responsibility for any errors or omissions, and make no warranty, express or implied, with respect to material contained within. For further information please refer to our Terms of Use.
